# The Effects of Weather Factors on Hand, Foot and Mouth Disease in Beijing

**DOI:** 10.1038/srep19247

**Published:** 2016-01-12

**Authors:** Weihua Dong, Xian’en Li, Peng Yang, Hua Liao, Xiaoli Wang, Quanyi Wang

**Affiliations:** 1 State Key Laboratory of Remote Sensing Science, Beijing Key Laboratory for Remote Sensing of Environment and Digital Cities & School of Geography and Remote Sensing Science, Beijing Normal University, China; 2 Institute for infectious disease and endemic disease control, Beijing Center for Disease Prevention and Control, China

## Abstract

The morbidity and mortality of hand, foot and mouth disease (HFMD) are increasing in Beijing, China. Previous studies have indicated an association between incidents of HFMD and weather factors. However, the seasonal influence of these factors on the disease is not yet understood, and their relationship with the enterovirus 71 (EV71) and Coxsackie virus A16 (CV-A16) viruses are not well documented. We analysed 84,502 HFMD cases from 2008 to 2011 in Beijing to explore the seasonal influence of weather factors (average temperature [AT], average relative humidity [ARH], total precipitation [TP] and average wind speed [AWS]) on incidents of HFMD by using a geographically weighted regression (GWR) model. The results indicated that weather factors differ significantly in their influence on HFMD depending on the season. AT had the greatest effect among the four weather factors, and while the influence of AT and AWS was greater in the summer than in the winter, the influence of TP was positive in the summer and negative in the winter. ARH was negatively correlated with HFMD. Also, we observed more EV71-associated cases than CV-A16 but there is no convincing evidence to show significant differences between the influences of the weather factors on EV71 and CV-A16.

Hand, foot and mouth disease (HFMD), a type of infectious disease mainly affecting children younger than 10 years of age, is occurring more and more frequently throughout the western Pacific in areas such as Japan, Malaysia, Singapore, Thailand, and China^1^,^2^,^3^,^4^. The annual report of the National Health and Family Planning Commission (NHFPC) shows that both incidence and death rates from HFMD are highest among class C infectious diseases in China^5^. It is reported that more than 7 million probable cases were reported to the China Center for Disease Control and Prevention during 2008–2012, of which 267, 942 were laboratory confirmed and 2,457 were fatal indicating an annual morbidity rate of 1.2/1000 and a fatality rate of 0.3/1000^6^. The public health threat caused by HFMD pushes the need to explore its risk factors and their mechanisms for better medical intervention.

HFMD is caused by various enteroviruses of which enterovirus 71 (EV71) and Coxsackie virus A16 (CV-A16) are the most frequently reported^7^. The clinical symptoms of HFMD include a painful buccal ulcerative lesion along with less painful skin rashes on the hands and feet, which may be associated with fever and lymphadenopathy^8^. It is mild and self-limiting but highly contagious. The incubation of HFMD may last 3–5 days^4^. The virus can spread from an infected person to others through either touching objects or surfaces that have the virus on them or close personal contact, such as kissing, hugging, coughing and sneezing. Unfortunately, the vaccine for HFMD is under development^9^ because the exact pathogenesis of HFMD in human infections are still unknown^10^, although multiple studies have been conducted on the pathology^11^,^12^,^13^, pharmacology^14^ and epidemiology^3^,^6^,^15^ of HFMD. Therefore, it is extremely important for public health departments to detect outbreaks and their spatial-temporal patterns in order to optimize the timely allocation of health resources.

Researchers have concentrated on the influence of weather factors on the occurrence of the disease. Hii *et al.*^16^ found a strong association of weekly temperature and rainfall with HFMD incidence in Singapore. Ma *et al.*^17^ examined the relationship between 13 meteorological parameters and HFMD occurrence in Hong Kong, finding that mean temperature, diurnal temperature differences, relative humidity and wind speed were positively associated with HFMD. This correlation was also confirmed in Guangzhou, Taiwan and Japan^18^,^19^,^20^. Although the influences of weather factors on HFMD are known to vary regionally, the potential effects of weather factors on HFMD are not well understood.

Recently, geographers and health researchers have collaborated to explore the relationship between disease and weather factors emphasizing on the spatial factors. For example, instead of using classical multivariate regression models that ignore spatial differences of HFMD incidences, Hu *et al.*^21^ employed the geographically weighted regression (GWR) model to investigate the associations between the child HFMD incidence and the selected factors (child population density, temperature, relative humidity and precipitation) at the county level in Mainland China. They found spatial heterogeneity of the strength and direction of association between these factors and the incidence of HFMD, which is confirmed by Bo *et al.*^22^. However, these studies did not address seasonal variance of the correlations.

This study aims to find seasonal and other potential effects of weather factors on HFMD using GWR model at sub-district level and monthly scale.

## Results

A total of 86, 037 inhabitant cases of hand, foot, and mouth disease were reported to the Beijing Centre for Disease Control and Prevention (Beijing CDC) surveillance system during January 1, 2008 to June 30, 2012 (average annual incidence, 93.90 per 10, 0000 person-years from 2008–2011), of which 2,967 (4.44%) were laboratory confirmed, 527 (0.61%) were severe and 7 (0.08‰) died. Among these records, 81,089 cases (95.96%) were 9 years old or younger. The male morbidity rate was higher than the female, with an average of male-to-female sex ratio of 1.46. Among the laboratory confirmed cases, 1297 (43.71%) were associated with EV71 and 1,142 (38.49%) were associated with CV-A16. CV-A16 and EV71 were the predominant pathogens accounting for 83.2% of the total sampled cases (Table 1). Among 7 deaths, 4 were associated with EV71 and 1 was associated with other enterovirus.

**Figure 1:**
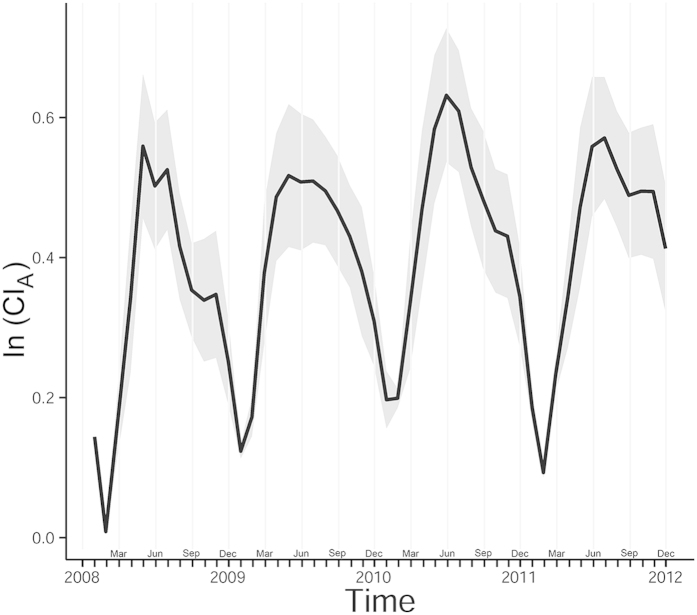
Average and standard deviation of monthly adjusted cumulative incidence (CI_A_). Note that the CI_A_ is log-transformed.

The general trend of the disease and the standard deviation of the adjusted cumulative incidence (CI_A_) is shown in Fig. 1. It can be seen that the incidence rate is higher in the summer than in other seasons, with the highest disease rate appearing from May to October.

**Figure 2:**
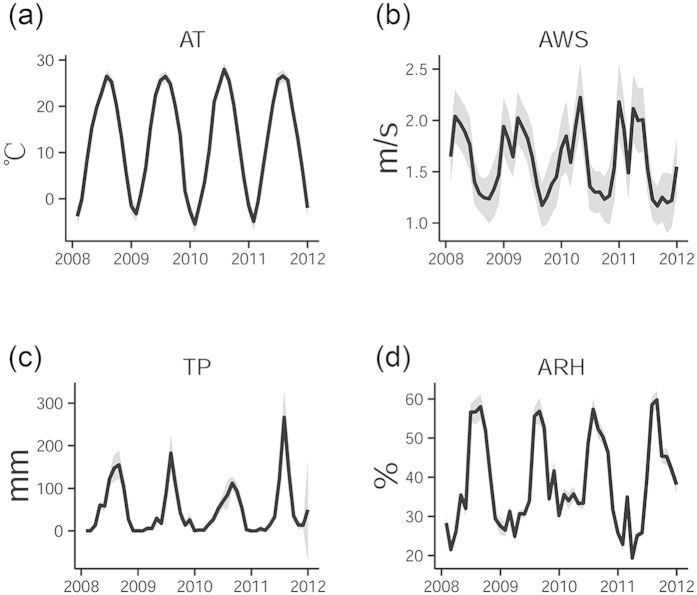
Monthly distribution of weather factors. (**a**) Average temperature; (**b**) Average wind speed; (**c**) Precipitation; and (**d**) Average relative humidity.

Figure 2 shows the monthly weather factors from 2008 to 2011. Wind speed in Beijing is greater in the winter and spring than in the summer and autumn; precipitation occurs almost exclusively in June, July and August; and humidity fluctuates more than other factors. Furthermore, temperature shows a normal trend, confirming the reliability of the interpolation result.

**Figure 3:**
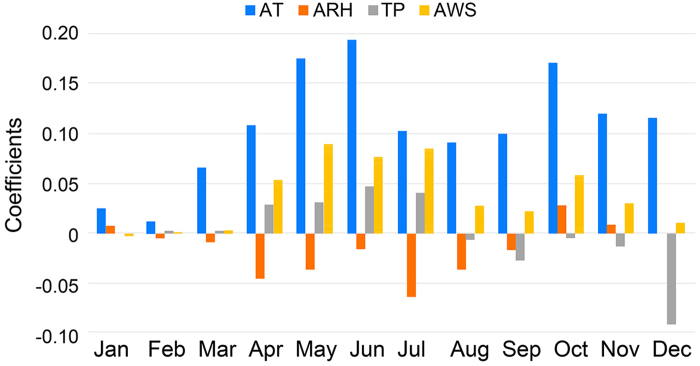
Average coefficients of weather factors. AT = average temperataure. ARH = average relative humidity. TP = total precipitation. AWS = average wind speed.

For each month from 2008 to 2011, the GWR model’s significance was tested by variance analysis (F tests), while the significance of estimated local parameters was checked with pseudo t-tests^23^. The results show that most models passed the F tests (Table S1). The models that did not pass primarily based on data for January, February and December, which have small sample sizes in these months. For the models that passed, however, each monthly model generated a set coefficient for the sub-district. These average coefficients of weather factors are shown in Fig. 3. Average temperature (AT) is shown to have a significantly higher sub-district coefficient than other factors, followed by average wind speed (AWS), total precipitation (TP) and average relative humidity (ARH) within the high disease rate period (usually April to August). Child population density (CPD) is not significantly related to HFMD in most time periods. The sub-district level and monthly scales analysis also allow for a more detailed analysis of seasonal relationships between HFMD and weather factors, which are is analysed in the following section.

Among all of the weather factors (Table S1), AT had the highest pass rate for statistical tests of significance, which is consistent with most research. AWS and TP coefficients were significant in specific months, and the mean R square value for the GWR models was 0.32. The significance of particular weather factors changes over the course of a typical year. According to Table S1, AT is the most stable, averaging 53.1% for most of the year. AWS is the most statistically significant in the winter, with an average of 28.3%, which may be because high wind speed is conducive to the spread of viruses. TP is the most statistically significant from May to July and has an overall average of 17.3%. Because precipitation in Beijing occurs almost exclusively from May to July, the average during these months increases to 32.9%. ARH is relatively randomly distributed at 9.8%.

## Discussion

### Seasonal pattern of influence of weather factors on HFMD

It is observed from Fig. 1 that two peaks occurred in 2008, 2010 and 2011 with the first peak appearing in June and the second in September to November while the disease in 2009 only peaked in May to July. It should also be noted that the second peak was far lower the first one. This is consistent with Xing *et al.*^6^ who found semiannual outbreaks based on full reported cases from 2008–2012 through the national surveillance system in China. Wang *et al.*^24^ suspected that the children transmitted the viruses to their younger siblings or neighbours at home and that the school opening of the autumn semester contributed to the second increase of the disease. Cao *et al.*^25^ argued that the intensive public health control measures during the summer months can strongly affect the occurrence of the disease. We also noted that during July 2009, the national surveillance system upgraded from a limited capacity to steady-state laboratory capacity which may introduce surveillance bias during the upgrade. However, the exact reasons to the single peak in 2009 and to the semiannual outbreaks in the other three years is still unclear.

It can be seen from Fig. 3 that over the course of a year, AT contributes most to HFMD among the four weather factors, followed by AWS, TP and ARH. AT and AWS are more influential in the summer than in the winter. In terms of TP, the influence is positive in the summer and negative in the winter, possibly because the winter precipitation in Beijing occurs mainly in the form of snow and inhibits outdoor activities. This variable should thus be used with caution in some model predictions.

**Figure 4:**
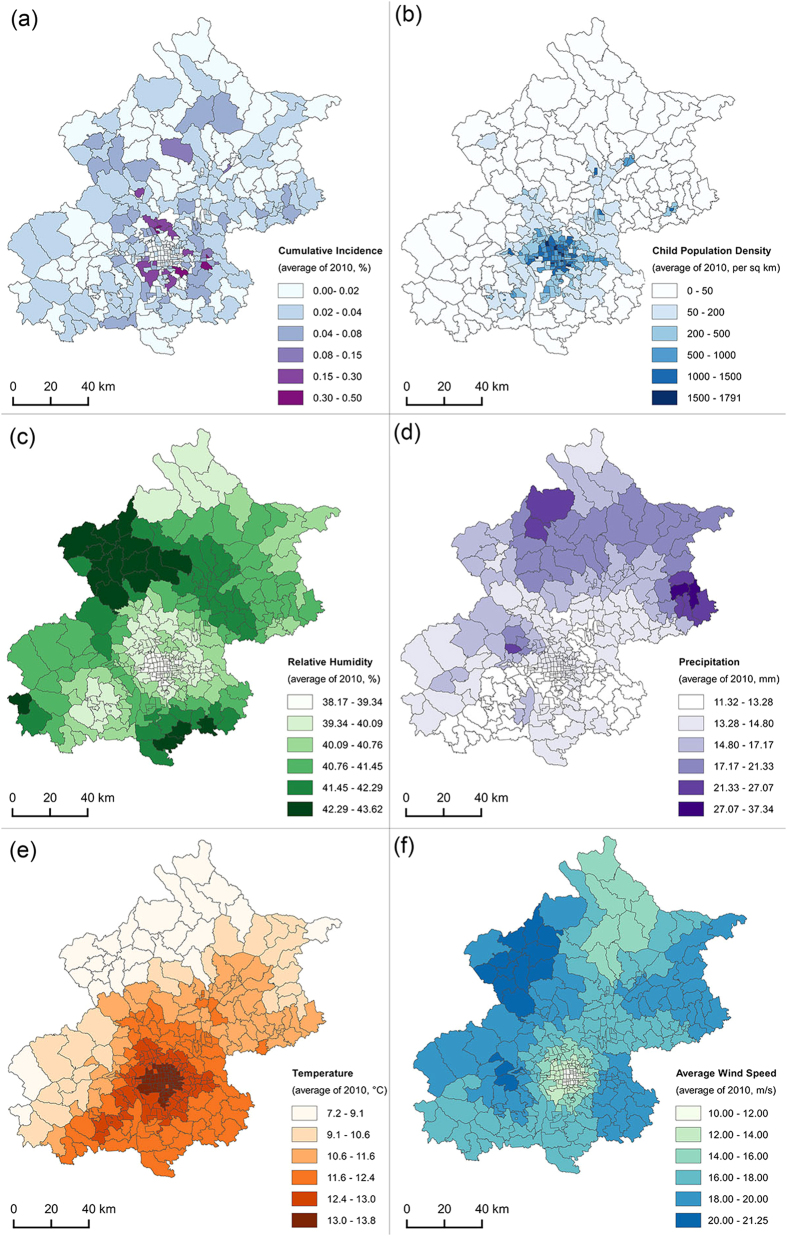
Spatial distribution of the yearly results of (**a**) HFMD cumulative incidence, (**b**) children population density, (**c**) relative humidity, (**d**) precipitation, (**e**) average temperature, and (f) average wind speed in 2010. Maps were generated using open source software QGIS 2.12 (http://www.qgis.org/).

The low passing rate of the model test of ARH (Table S1) indicates that that it is a weak predictor of seasonal HFMD patterns. Moreover, it is observed from Fig. 3 that the ARH is negatively associated with the HFMD incidence in most of the months, which is consistent with previous studies that the latitude of the study area is similar to Beijing such as Shandong Province^26^. In contrast, previous studies regarding to lower latitude areas such as Hong Kong^17^, Taiwan^18^ and Japan^20^ found positive association of relative humidity. A further look into the spatial distributions of ARH and CI_A_ (Fig. 4) reveals that they have a negative spatial relationship. The high ARH values are focused on rural areas, while the high CI_A_ values are primarily distributed in urban areas of Beijing. Relative to rural areas, the low ARH of urban areas could be influenced by Beijing’s urban heat island^27^.

**Figure 5:**
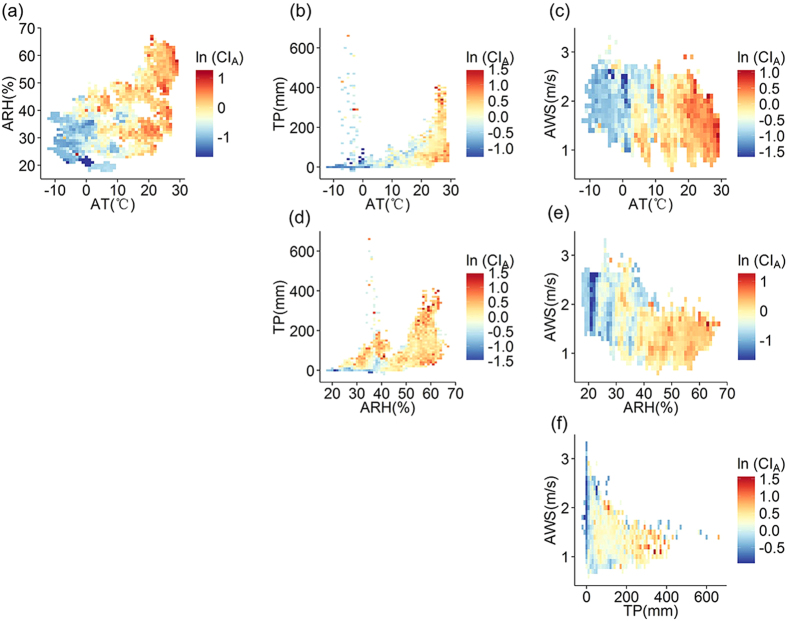
Mean cumulative incidence driven by weather factor (AT, ARH, TP, AWS) combinations from 2008 to 2011.

The mechanism relationship between weather factors and HFMD is complex. We further visualized the relationship between any two weather factors and the cumulative incidence of the disease (Fig. 5) to explore the potential patterns of interactive influence driven by combined weather factors. Four notable observations can be found. First, the combinations of average temperature and any other weather factor show positive relationships (Fig. 5a–c). Second, although the ARH-AT combination shows a positive relationship with disease, the ARH-TP/ARH-AWS combination shows that this relationship is not consistent. Third, both the TP-AT and TP-ARH combinations have two peaks (Fig. 5b,d). One is a low-AT/ARH and high-TP peak and the other is a high-AT/ARH and high-TP peak. The former mainly occurs in the winter, and the latter occurs in the summer since average temperature is lower in winter months than in summer months. TP is positively related to the disease in the summer and negatively related in the winter, as demonstrated in Fig. 3. Finally, the AWS-other combinations (Fig. 5c,e,f) do not show an obvious positive or negative relationship with disease. In general, the most stable variable is AT, while other weather factors do not show a stable relationship with the disease.

### Comparison of EV71 and CV-A16

It is revealed from the annual incidence (Table 1) that EV71 predominantly circulated during 2008 and 2010 with co-circulation of CV-A16, while CV-A16 predominantly circulated during 2009 and 2011 with co-circulation of EV71. This annual co-circulation of EV71 and CV-A16 in China^28^ differs from other outbreaks such as a 3-year epidemic cycle of EV71 with co-circulation of CV-A16 in Malaysia and 3-year predominance of EV71 in Japan^1^. It is possible that patients can develop immunity to the predominant virus but the immune system cannot prevent infection from other serotypes of enterovirus^7^, resulting in the cyclic epidemic of EV71 and CV-A16.

It is reported that EV71 infection can cause severe central nervous system complications and fatal pulmonary oedema^12^, and that EV71 was more commonly associated with more severe and fatal cases while CV-A16 resulted in milder and lower incidence of neurological disease^24^,^29^. It is generally acknowledged that EV71 infection is much severer than CV-A16 clinically^6^,^29^. In this study, more EV71 cases were observed than CV-A16 both across age groups except < 1 year and 2–3 years (Figure S2), and across months except March, April, November and December (Figure S3).

To explore potential difference of the influence of weather factors on EV71 and CV-A16, we compared the number of cases associated with EV71, CV-A16 and other enterovirus using a chi-square test for each month (Table S2). It can be seen that the EV71 was mostly predominant in the summer months, while the CV-A16 was predominant in both summer and winter months. The EV71 was predominant from May to July in 2008. The influence of AT, TP and AWS was positive, but that of ARH was negative. In 2009, a year in which CV-A16 was predominant, the ARH was positive in May and negative in June and August, while other factors were similar to those of 2008. The dominance of EV71 was very similar in 2008 and 2010. No sub-district level of ARH and TP were statistically significant in October. There was only one month (May) when EV71 was predominant in the summer of 2011. The CV-A16 virus became predominant in November and December of that year. TP was positive in November and negative in December, and AWS was generally positive.

In addition, a correlation analysis between the weather factors and the enterovirus was performed (Figure S5). It is observed that AT was positively correlated to EV71 with a Pearson correlation coefficient of 0.572 (R^2^ = 0.32) which is higher than CV-A16 (coefficient = 0.490, R^2^ = 0.24), and that TP was also positively associated with EV71 and CV-A16 with coefficients of 0.375 and 0.314, respectively. But neither AWS nor ARH had a significant association with the two viruses.

To sum up, it seems that AT and TP had a stronger relationship to EV71 than CV-A16 but there is no convincing evidence to conclude that significant differences exist between the influences of the weather factors on EV71 and on CV-A16. The results need further investigation because we were limited by the small sample size of laboratory-confirmed cases (N = 2,967, distributed on 319 sub-districts during 12 months × 4 years) to conduct an exhaustive subgroup experiment to compare the differences between the two viruses, which was also beyond the scope of the study.

### Cross correlation validation

In order to cross check the correlation of HFMD incidence with the weather factors, we used cross-correlation function (CCF)^30^ to detect lags of the weather variables that might be useful predicators of HFMD outbreaks and the results is presented in Figure S6. Although the autocorrelation function (ACF) values from lag = −12 to 12 months were calculated, we focused on a shorter duration because a too long time lag (e.g., ≥ 6 months) seems unreasonable. In addition, time lags of less than 8 weeks were commonly reported in previous studies^16^,^17^,^26^ thus we searched peak ACF values between lag = −2 to 2 months for further analysis.

It can be seen that for average temperature and monthly precipitation, ACF peaks at lag = 0 month (ACF = 0.75) and lag = 0 or −1 month (ACF = 0.45), respectively. This indicates that the association between these two weather factors and HFMD incidence occurred at time lag within one month. This is consistent with previous studies^16^,^17^ that found a two weeks’ time lag. It is also observed that the peak ACF value of temperature is positive meaning that an increasing temperature is likely to lead to an increasing HFMD incidence.

It is interesting to note that average wind speed was negatively associated with HFMD infections at a time lag = −2 months (ACF = −6.5), which is in contrast with our earlier results and previous evidence. In terms of relative humidity, it is observed that ACF peaked at lag = −1 month (ACF = 6.5). Combined with the earlier finding that relative humidity was negative in summer months and positive in winter months (Figure 3), we speculate that the positive influence of relative humidity on HFMD might be more significant^17^,^26^.

To understand exact kinetic mechanisms for the relationship between meteorological parameters and HFMD require further investigation. While weather factors do not influence the disease directly, we speculate that there are at least two ways that they can play a role. First, weather factors can affect the external environment thereby mediating biological activity, propagation and transmission of the enterovirus. For example, wind can promote air pollutants where the enterovirus can survive and thus accelerate the spread of HFMD virus^26^. In addition, a recent laboratory study revealed that enteroviruses are very resilient to the environmental conditions of the gastrointestinal tract, and that their stability relies on the temperature, humidity and UV radiation^31^. Similar dependence of influenza virus on weather factors was also reported by Lowen *et al.*^32^.

Second, weather factors influence individual’s physical activity^33^. It is stated that an increase of rainfall can lower physical activity while an increase of temperature can promote activity rates, and that physical activity of adolescence was lower during winter and higher during warmer months^34^. The variance of physical activity influenced by weather conditions plays an important role in transporting virus by close personal contact, to some extent accounting for the seasonal variance of HFMD occurrence. Although the complexity of HFMD cannot be fully explained by weather factors, our results provide new quantitative evidence indicating the influence of weather factors on HFMD infections at a finer spatial-temporal scale.

## Methods

### Ethics statement

This research was approved by the Institutional Review Board at the Beijing CDC, and the methods were carried out in accordance with the principles of the Declaration of Helsinki. All records were anonymized and no individual information can be identified.

### Data and preprocessing

The following three types of data were obtained and preprocessed:

1. HFMD incidence data. A total of 86,037 daily records of HFMD of Beijing (Figure S1) from January 1, 2008 to June 30, 2012 were obtained from the Beijing CDC. Each record contains the patient address (mostly formatted as ‘city + district + sub-district + street + number’), age and date of onset. Of these, 84,502 records (98.22%) were successfully geocoded to the sub-district level and the others were excluded from analysis. Then the data were aggregated to monthly level in order to avoid a shortage of small samples in the analysis. Monthly time scale was also consistent with the typical period of HFMD incubation, disease and cure. The incidence data were further processed into cumulative incidence which is described later.
2. Demographic data. Child population data were obtained from the State Statistics Bureau. Variation in population size in years was normalized. Then child population density (CPD) was calculated as child population (aged 0–9) divided by district area. The spatial distribution of CPD of an example year (2010) is shown in Figure 4(b).
3. Meteorology data. Daily data for four weather factors—average temperature (AT), average wind speed (AWS), total precipitation (TP) and average relative humidity (ARH) —were obtained from the Beijing Climate Centre. The data were recorded by 280 spatially distributed meteorology stations including 20 manual and 260 automatic stations. We used Kriging^35^ interpolation method which was implemented in ESRI ArcGIS Spatial Analyst tools module to get daily values of the four weather variables with a spatial resolution of 500 meters in raster format. Then the monthly data of AT, ARH, TP and AWS of each sub-district were obtained by calculating the average values within corresponding sub-districts. These four weather variables and the above mentioned CPD were the independent variables in the GWR model. The spatial distribution of these four factors of an example year (2010) is shown in Fig. 4(c–f).

### Cumulative incidence inference of HFMD

Using the absolute number of HFMD incidences is inappropriate for modeling its association with weather factors because the number of incidences is related to the total child population. Therefore, the absolute number of cases was normalized using the cumulative incidence (CI)^19^ which is defined by the ratio of the number of incident cases and the total population aged 0–9 years. The CI reflects the probability of disease occurrence in a given place, with larger populations having more stable CI values.

It is important to note that a zero-CI value is unlikely to appear, though some months in the dataset have no incidences at the sub-district level. This is likely due to small sample size, random effects, and other data variations such as collection bias or geocoding bias. To avoid the problem, a hierarchical Bayes model, the Besag, York, and Mollie (BYM) model, was introduced to reduce the CI’s spatial variance^36^,^37^,^38^. The model was solved by an MCMC simulation in WinBUGS 1.4, and the length of burn-in sequence was 4000^21^. The output of the model which is termed as adjusted cumulative incidence inference (CI_A_) was regarded as the dependent variable in GWR model. The spatial distribution of CI_A_ of an example year (2010) is shown in Fig. 4(a).

### Geographically weighted regression

The GWR model was used to explore the relationship between the five independent variables (AT, ARH, TP, AWS and CPD) and the dependent variable (CI_A_). The GWR model is a classic approach to spatial analysis, and extends a transitional regression framework by allowing parameters to be estimated locally, so the model can be expressed as (1)^23^.


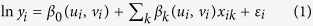


where *(u*_*i*_*, v*_*i*_) denotes the coordinates at the *i*_th_ point in space and *β*_*k*_*(u*_*i*_*, v*_*i*_) is the value of the continuous function *β*_*k*_*(u, v)* at point *i*. In this study, *y* is the log-transformed dependent variable CI_A_. *x*_*i*_ is the independent variable, including CPD and four weather factors, while *β*_*ik*_ are local regression parameters to be estimated. Compared with classical regression models, GWR embeds spatial information into the regression model. Further details on the methods can be found in Fotheringham’s work^23^. In this study, the method implemented in ESRI ArcGIS and GWR4 was adopted.

## Additional Information

**How to cite this article**: Dong, W. *et al.* The Effects of Weather Factors on Hand, Foot and Mouth Disease in Beijing. *Sci. Rep.* **6**, 19247; doi: 10.1038/srep19247 (2016).

## Supplementary Material

Supplementary Information

## Figures and Tables

**Table 1:** Summary of laboratory confirmed cases.

	2008		2009		2010		2011	
	Cases	Percent	Cases	Percent	Cases	Percent	Cases	Percent
*CV-A16*	39	15.4%	279	52.6%	381	30.9%	443	46.6%
*EV71*	186	73.2%	185	34.9%	564	45.8%	362	38.1%
*Other*	29	11.4%	66	12.5%	287	23.3%	146	15.4%
*Total*	254	100.0%	530	100.0%	1232	100.0%	951	100.0%
