# Supplementary material for: The Effects of Weather Factors on Hand, Foot and Mouth Disease in Beijing
Source: Sci Rep. 2016 Jan 12;6:19247. doi: 10.1038/srep19247 (PMC4709685; doi:10.1038/srep19247)
Supplement: Supplementary Information [file srep19247-s1.doc]

The Effects of Weather Factors on Hand, Foot and Mouth Disease in Beijing

Weihua Dong, Xian’en Li, Peng Yang , Hua Liao, Xiaoli Wang, Quanyi Wang

**Supplementary Table S1. Descripted statistics for the GWR model results**

| Month | R^2^ | Intercept | CPD | AWS | TP | ARH | AT |
| --- | --- | --- | --- | --- | --- | --- | --- |
| 200801 | 0.35** | 100.0% | 0.0% | 88.4% | 74.0% | 27.0% | 63.6% |
| 200802 | 0.71** | 100.0% | 30.7% | 90.6% | 0.0% | 0.0% | 43.3% |
| 200803 | 0.10** | 100.0% | 0.0% | 0.0% | 0.0% | 0.0% | 10.0% |
| 200804 | 0.65** | 100.0% | 0.0% | 87.1% | 42.0% | 0.9% | 85.9% |
| 200805 | 0.36** | 100.0% | 0.0% | 59.9% | 1.3% | 0.0% | 72.1% |
| 200806 | 0.25** | 100.0% | 0.0% | 7.5% | 99.7% | 0.0% | 88.7% |
| 200807 | 0.40** | 100.0% | 0.0% | 74.3% | 49.5% | 0.0% | 29.8% |
| 200808 | 0.12** | 100.0% | 0.0% | 23.2% | 35.4% | 0.0% | 0.0% |
| 200809 | 0.32** | 100.0% | 0.0% | 4.7% | 0.0% | 0.0% | 32.0% |
| 200810 | 0.67** | 100.0% | 0.0% | 10.7% | 0.0% | 89.0% | 57.1% |
| 200811 | 0.28** | 100.0% | 0.0% | 0.0% | 1.6% | 0.0% | 36.1% |
| 200812 | 0.19** | 100.0% | 0.0% | 10.3% | 0.0% | 1.6% | 53.0% |
| 200901 | 0.01 | - | - | - | - | - | - |
| 200902 | 0.04 | - | - | - | - | - | - |
| 200903 | 0.24** | 100.0% | 0.0% | 0.0% | 0.0% | 0.0% | 31.0% |
| 200904 | 0.23** | 100.0% | 0.0% | 1.6% | 0.6% | 0.0% | 14.7% |
| 200905 | 0.40** | 100.0% | 0.0% | 16.6% | 65.2% | 42.0% | 74.3% |
| 200906 | 0.44** | 100.0% | 0.0% | 81.2% | 39.8% | 0.0% | 76.2% |
| 200907 | 0.33** | 100.0% | 0.0% | 30.1% | 2.5% | 6.9% | 68.3% |
| 200908 | 0.15** | 100.0% | 0.0% | 0.0% | 0.0% | 0.0% | 27.6% |
| 200909 | 0.12** | 100.0% | 0.0% | 17.9% | 18.8% | 0.0% | 54.2% |
| 200910 | 0.29** | 100.0% | 0.0% | 3.1% | 0.0% | 0.0% | 53.0% |
| 200911 | 0.57** | 100.0% | 0.0% | 32.6% | 0.0% | 18.2% | 55.5% |
| 200912 | 0.14** | 100.0% | 0.0% | 0.0% | 0.0% | 0.0% | 37.0% |
| 201001 | 0.16 | - | - | - | - | - | - |
| 201002 | 0.17 | - | - | - | - | - | - |
| 201003 | 0.60** | 100.0% | 0.0% | 19.1% | 46.7% | 19.7% | 52.7% |
| 201004 | 0.51** | 100.0% | 0.0% | 6.3% | 19.7% | 0.0% | 57.1% |
| 201005 | 0.43** | 100.0% | 0.0% | 66.8% | 61.4% | 0.0% | 56.7% |
| 201006 | 0.28** | 100.0% | 0.0% | 28.8% | 0.0% | 15.4% | 56.7% |
| 201007 | 0.15** | 100.0% | 0.0% | 14.4% | 7.8% | 0.0% | 64.9% |
| 201008 | 0.35** | 100.0% | 0.0% | 21.9% | 0.0% | 8.2% | 44.8% |
| 201009 | 0.20** | 100.0% | 0.0% | 22.3% | 0.0% | 1.6% | 24.1% |
| 201010 | 0.08* | 100.0% | 0.0% | 85.0% | 10.7% | 0.0% | 54.9% |
| 201011 | 0.32** | 100.0% | 0.0% | 27.6% | 0.0% | 55.2% | 49.5% |
| 201012 | 0.05 | - | - | - | - | - | - |
| 201101 | 0.02 | - | - | - | - | - | - |
| 201102 | 0.38** | 100.0% | 0.0% | 0.0% | 0.0% | 4.7% | 37.0% |
| 201103 | 0.09 | - | - | - | - | - | - |
| 201104 | 0.34** | 100.0% | 0.0% | 1.6% | 13.2% | 0.0% | 43.6% |
| 201105 | 0.52** | 100.0% | 0.0% | 76.5% | 0.0% | 32.9% | 65.5% |
| 201106 | 0.45** | 100.0% | 0.0% | 33.5% | 40.1% | 22.6% | 90.9% |
| 201107 | 0.28** | 100.0% | 0.0% | 94.0% | 27.9% | 0.0% | 38.6% |
| 201108 | 0.27** | 100.0% | 0.0% | 0.0% | 0.0% | 0.0% | 70.5% |
| 201109 | 0.35** | 100.0% | 0.0% | 1.6% | 0.0% | 4.7% | 62.1% |
| 201110 | 0.38** | 100.0% | 0.0% | 8.8% | 18.5% | 37.3% | 66.8% |
| 201111 | 0.30** | 100.0% | 0.0% | 11.6% | 31.3% | 19.4% | 54.2% |
| 201112 | 0.21** | 84.3% | 0.0% | 5.6% | 2.8% | 0.0% | 72.4% |

***Note****:‘*********’ : p<0.05, ‘**********’: p<0.01*

**Supplementary Table S2. Chi-square test of EV-71, CV-A16 and other viruses.**

|  | EV71 | CA-V16 | Other | EV71&CA-V16&Other | | | EV71&CA-V16 | | | Dominant  Virus |
| --- | --- | --- | --- | --- | --- | --- | --- | --- | --- | --- |
|  |  |  |  | chi-square | df | p-value | chi-square | df | p-value |  |
| 200801 | - | - | - | - | - | - | - | - | - | None |
| 200802 | - | - | - | - | - | - | - | - | - | None |
| 200803 | - | - | - | - | - | - | - | - | - | None |
| 200804 | 5 | 10 | 0 | 1.667 | 1 | 0.197 | 1.667 | 1 | 0.197 | None |
| 200805 | 221 | 50 | 36 | 207.368 | 2 | 0.000** | 107.9 | 1 | 0.000** | EV71 |
| 200806 | 98 | 13 | 6 | 134.513 | 2 | 0.000** | 65.09 | 1 | 0.000** | EV71 |
| 200807 | 15 | 3 | 0 | 8 | 1 | 0.005** | 8 | 1 | 0.005** | EV71 |
| 200808 | 8 | 0 | 1 | 5.444 | 1 | 0.02** | - | - | - | None |
| 200809 | - | - | - | - | - | - | - | - | - | None |
| 200810 | 0 | 0 | 2 | - | - | - | - | - | - | None |
| 200811 | 0 | 0 | 2 | - | - | - | - | - | - | None |
| 200812 | 0 | 0 | 1 | - | - | - | - | - | - | None |
| 200901 | - | - | - | - | - | - | - | - | - | None |
| 200902 | 0 | 0 | 1 | - | - | - | - | - | - | None |
| 200903 | 36 | 0 | 5 | 23.439 | 1 | 0.000** | - | - | - | None |
| 200904 | 75 | 76 | 16 | 42.407 | 2 | 0.000** | 0.007 | 1 | 0.935 | None |
| 200905 | 63 | 89 | 18 | 45.541 | 2 | 0.000** | 4.447 | 1 | 0.035* | CA-V16 |
| 200906 | 31 | 72 | 11 | 50.895 | 2 | 0.000** | 16.32 | 1 | 0.000** | CA-V16 |
| 200907 | 46 | 62 | 17 | 24.976 | 2 | 0.000** | 2.37 | 1 | 0.124 | None |
| 200908 | 22 | 46 | 12 | 22.9 | 2 | 0.000** | 8.471 | 1 | 0.004** | CA-V16 |
| 200909 | 14 | 24 | 8 | 8.522 | 2 | 0.014* | 2.632 | 1 | 0.105 | None |
| 200910 | 14 | 17 | 6 | 5.243 | 2 | 0.073 | 0.29 | 1 | 0.59 | None |
| 200911 | 10 | 25 | 8 | 12.047 | 2 | 0.002** | 6.429 | 1 | 0.011* | CA-V16 |
| 200912 | 5 | 17 | 5 | 10.667 | 2 | 0.005** | 6.545 | 1 | 0.011* | CA-V16 |
| 201001 | 1 | 5 | 3 | 2.667 | 2 | 0.264 | 2.667 | 1 | 0.102 | None |
| 201002 | 5 | 4 | 3 | 0.5 | 2 | 0.779 | 0.111 | 1 | 0.739 | None |
| 201003 | 32 | 47 | 10 | 23.348 | 2 | 0.000** | 2.848 | 1 | 0.091 | None |
| 201004 | 75 | 90 | 48 | 12.761 | 2 | 0.002 | 1.364 | 1 | 0.243 | None |
| 201005 | 177 | 137 | 77 | 38.875 | 2 | 0.000** | 5.096 | 1 | 0.024* | EV71 |
| 201006 | 202 | 96 | 140 | 38.849 | 2 | 0.000** | 37.705 | 1 | 0.000** | EV71 |
| 201007 | 239 | 80 | 71 | 137.4 | 2 | 0.000** | 79.251 | 1 | 0.000** | EV71 |
| 201008 | 87 | 42 | 26 | 38.723 | 2 | 0.000** | 15.698 | 1 | 0.000** | EV71 |
| 201009 | 58 | 58 | 28 | 12.5 | 2 | 0.002** | 0 | 1 | 1 | None |
| 201010 | 48 | 35 | 16 | 15.697 | 2 | 0.000** | 2.036 | 1 | 0.154 | None |
| 201011 | 40 | 28 | 16 | 10.286 | 2 | 0.006** | 2.118 | 1 | 0.146 | None |
| 201012 | 24 | 4 | 9 | 17.568 | 2 | 0.000** | 14.286 | 1 | 0.000** | EV71 |
| 201101 | 2 | 1 | 1 | 0.5 | 2 | 0.779 | 0.333 | 1 | 0.564 | None |
| 201102 | 5 | 0 | 0 | - | - | - | - | - | - | None |
| 201103 | 6 | 15 | 10 | 3.935 | 2 | 0.14 | 3.857 | 1 | 0.05* | None |
| 201104 | 24 | 18 | 11 | 4.792 | 2 | 0.091 | 0.857 | 1 | 0.355 | None |
| 201105 | 103 | 67 | 42 | 26.613 | 2 | 0.000** | 7.624 | 1 | 0.006** | EV71 |
| 201106 | 124 | 105 | 66 | 17.783 | 2 | 0.000** | 1.576 | 1 | 0.209 | None |
| 201107 | 103 | 82 | 29 | 40.776 | 2 | 0.000** | 2.384 | 1 | 0.123 | None |
| 201108 | 76 | 58 | 15 | 39.557 | 2 | 0.000** | 2.418 | 1 | 0.12 | None |
| 201109 | 73 | 47 | 13 | 40.842 | 2 | 0.000** | 5.633 | 1 | 0.018* | EV71 |
| 201110 | 46 | 63 | 4 | 48.973 | 2 | 0.000** | 2.651 | 1 | 0.103 | None |
| 201111 | 23 | 112 | 5 | 140.671 | 2 | 0.000** | 58.674 | 1 | 0.000** | CA-V16 |
| 201112 | 8 | 35 | 5 | 34.125 | 2 | 0.000** | 16.953 | 1 | 0.000** | CA-V16 |

***Note****:‘*********’ : p<0.05, ‘**********’: p<0.01*


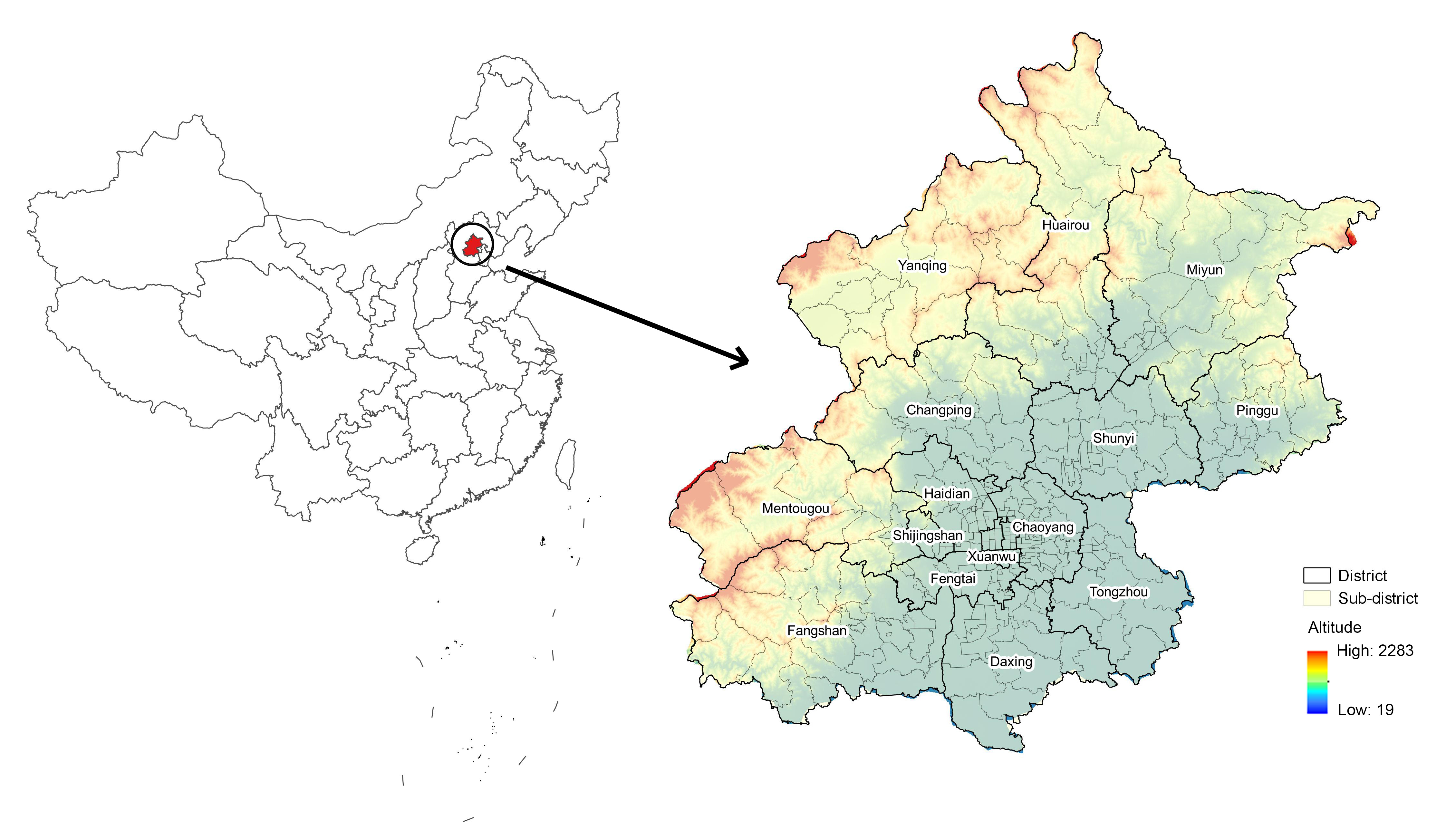


**Supplementary Figure S1**. Location and administrative divisions of districts and sub-districts of Beijing. Beijing is the capital city of China located in northern China (Figure S1). It is centered at 39°54′20″N 116°25′29″E with an area of 16,800 km2 and a population of over 20 million. Beijing consists of 16 districts divided into 319 sub-districts.


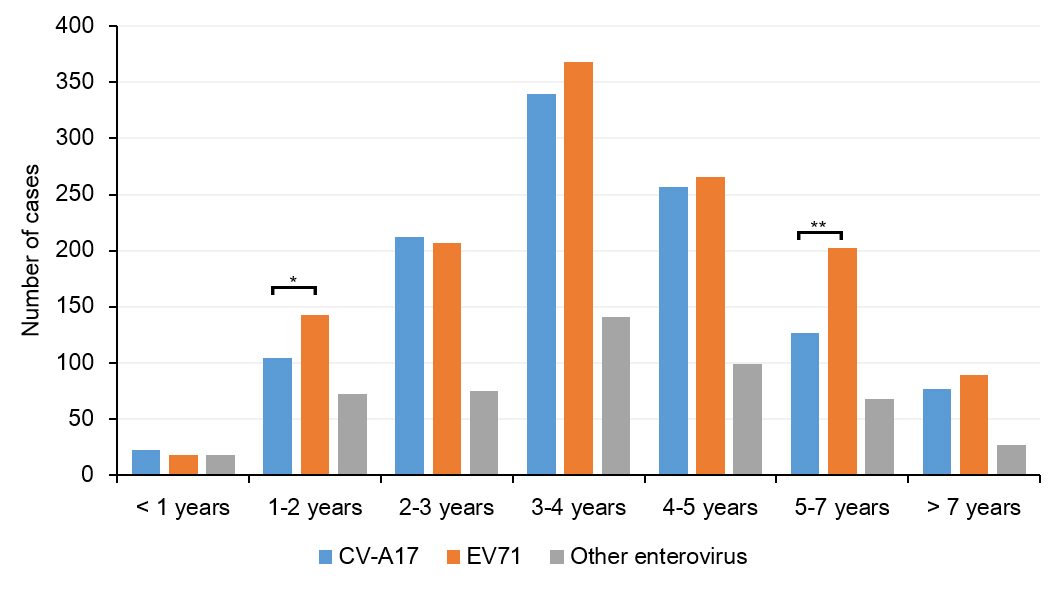


***Note****:‘*********’ : p<0.05, ‘**********’: p<0.01*

**Supplementary Figure S2**. Age distribution of laboratory-confirmed cases of hand, foot, and mouth disease in Beijing, 2008-11.


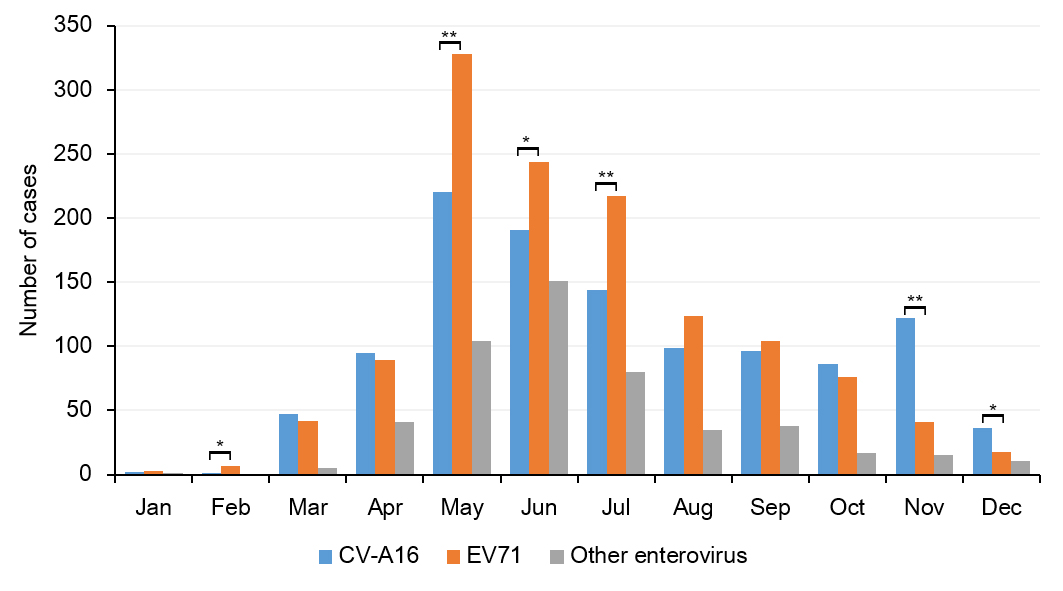


***Note****:‘*********’ : p<0.05, ‘**********’: p<0.01*

**Supplementary Figure S3**. Monthly distribution of laboratory-confirmed cases of hand, foot, and mouth disease in Beijing, 2008-11.


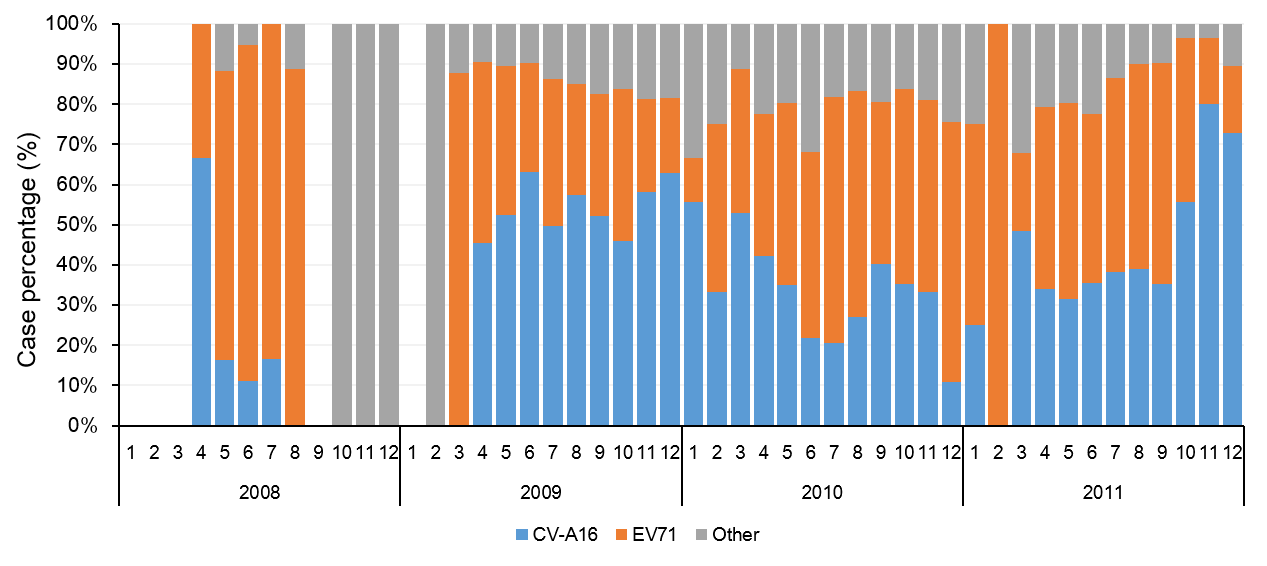


**Supplementary Figure S4**. Monthly distribution of case percentage of CV-A16, EV71 and other enteroviruses.


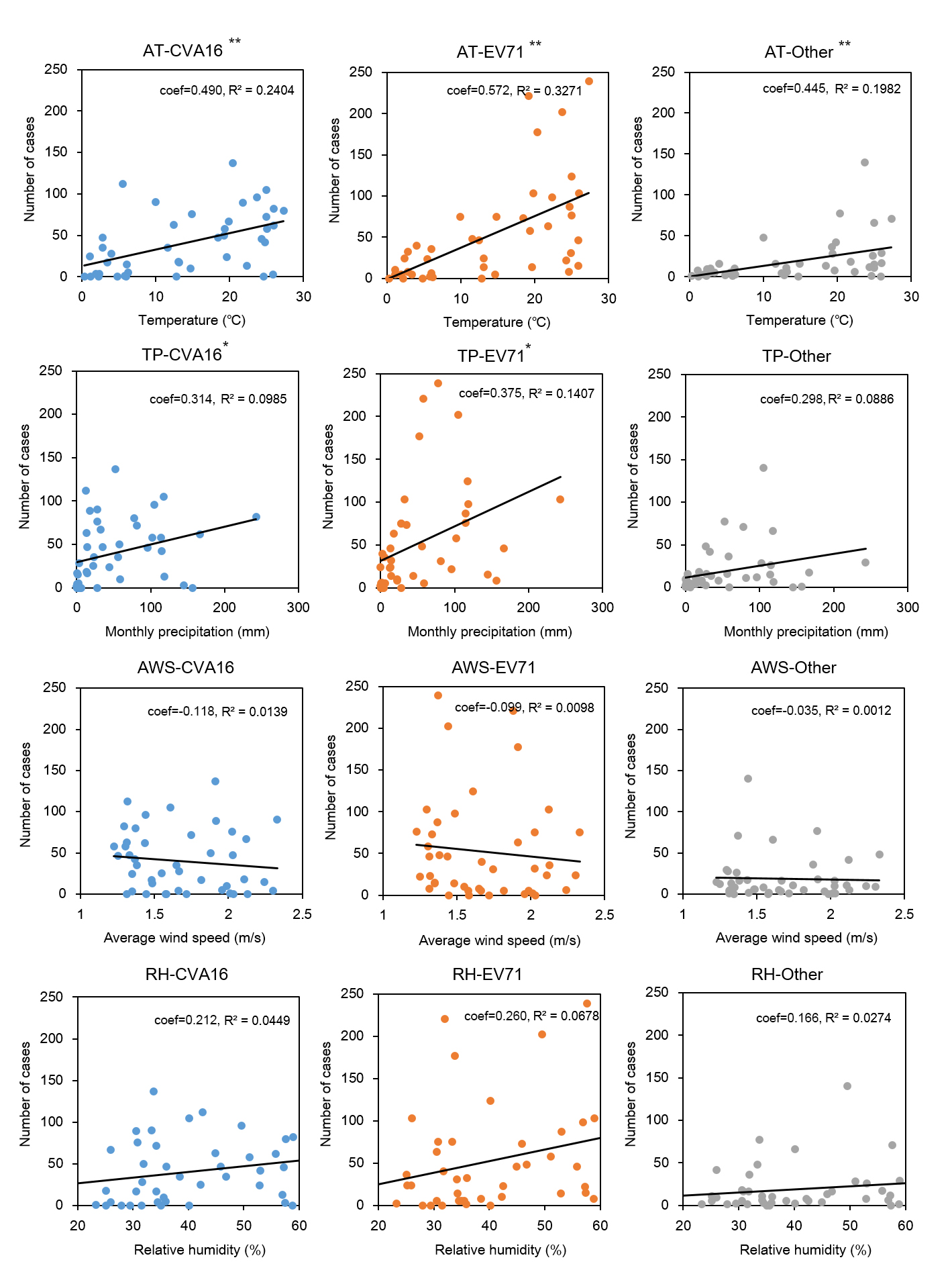


***Note****:‘*********’ : p<0.05, ‘**********’: p<0.01*

**Supplementary Figure S5**. Scatter plot of weather factors (AT, AWS, ARH, and TP) and HFMD enteroviruses (CV-A16, EV71, and other enteroviruses). AT=average temperature. AWS=average wind speed. ARH=average relative humidity. TP=total precipitation. Coef= Pearson correlation coefficient.


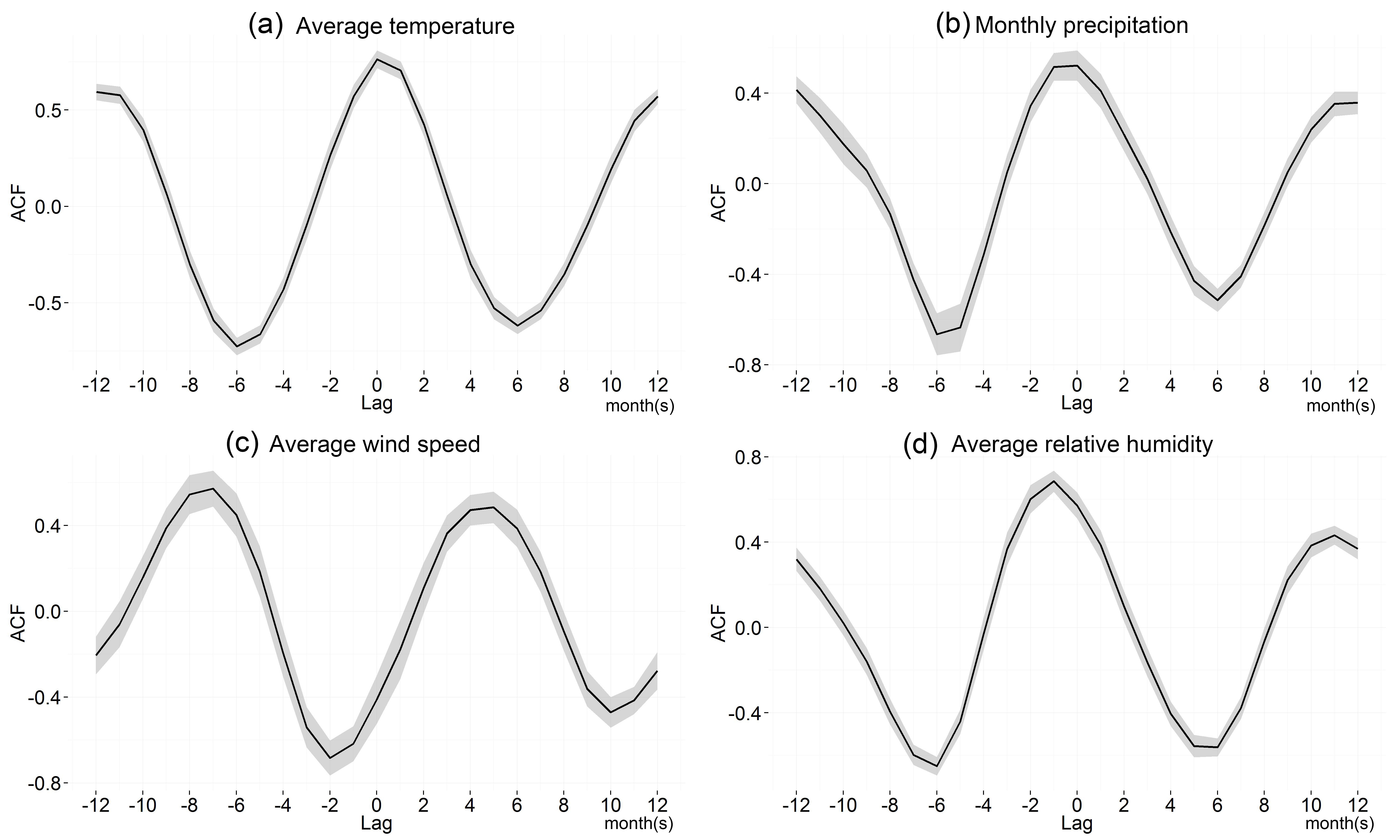


**Supplementary Figure S6**. Monthly cross correlation of weather factors and HFMD incidence. (a) Average temperature. (b) Monthly precipitation. (c) Average wind speed. (d) Average relative humidity. ACF= sample autocorrelation function.
